# MiR-199a-5p–HIF-1α-STAT3 Positive Feedback Loop Contributes to the Progression of Non-Small Cell Lung Cancer

**DOI:** 10.3389/fcell.2020.620615

**Published:** 2021-02-18

**Authors:** Xingping Yang, Yuzhen Zheng, Jian Tan, Renjiang Tian, Piao Shen, Weijie Cai, Hongying Liao

**Affiliations:** ^1^Department of Thoracic Surgery, Thoracic Cancer Center, The Sixth Affiliated Hospital of Sun Yat-sen University, Guangzhou, China; ^2^Department of Thoracic Surgery, Affiliated Cancer Hospital & Institute of Guangzhou Medical University, Guangzhou, China

**Keywords:** MiR-199a-5p, HIF-1α, stat3, non-small cell lung cancer, bevacizumab

## Abstract

**Background:** Non-small cell lung cancer (NSCLC) is the most common malignancy worldwide. MiR-199a-5p has been reported to play important roles in multiple tumors, inclusive of NSCLC. However, little is definitively known pertaining to its explicit mechanism of action in NSCLC.

**Methods:** The expressions of miR-199a-5p and hypoxia-inducible factor-1α (HIF-1α) mRNA were quantified employing qRT-PCR. H1299 and A549 cells were transiently transfected with miR-199a-5p mimics or inhibitors. Then, CCK-8 assays, flow cytometry analysis, and Transwell assay were performed for detecting cell proliferation, cell cycle, apoptosis, migration, and invasion of NSCLC cells, respectively. HIF-1α, signal transducer and activator of transcription 3 (STAT3), and p-STAT3 expressions were detected via Western blotting. Bioinformatic analysis and dual-luciferase assay were performed to investigate the interactions among miR-199a-5p, HIF-1α, and STAT3. Xenograft models were established with nude mice for further analyzing the bevacizumab resistance of NSCLC cells.

**Results:** MiR-199a-5p expression was markedly attenuated in NSCLC tissues and cell lines. Overexpression of miR-199a-5p repressed the proliferation, migration, and invasion but induced the apoptosis of NSCLC cells. HIF-1α was identified as a direct target of miR-199a-5p. There was a positive feedback loop among miR-199a-5p, HIF-1α, and STAT3. Co-transfection of HIF-1α or STAT3 overexpression plasmids counteracted the effects of miR-199a-5p. *In vivo* experiments indicated that the feedback loop was in association with the bevacizumab resistance of NSCLC cells.

**Conclusion:** MiR-199a-5p blocked the progression of NSCLC and sensitized NSCLC cells to bevacizumab by suppressing HIF-1α and STAT3, while the HIF-1α/STAT3 axis suppressed the expression of miR-199a-5p, which forms a positive feedback loop to promote the sustaining progression of NSCLC.

## Introduction

Lung cancer is the most common and leading cause of cancer-related deaths worldwide, among which non-small cell lung cancer (NSCLC) accounts for more than 80% of cases (Bradbury et al., 2017). Even though novel therapeutic approaches for NSCLC have significantly improved the prognosis of patients, almost all of the current approved drugs face the problem of drug resistance (Brody et al., 2017; Yu et al., 2018; Zheng et al., 2018). It is still necessary to further clarify the mechanism of sustaining progression of NSCLC, which may provide clues for developing novel therapy strategies to improve the survival time of patients.

Non-coding RNAs, including microRNAs (miRNAs), participate in the pathogenesis of diverse human diseases, inclusive of NSCLC (Tu et al., 2016). MiR-199a-5p, markedly downregulated in NSCLC tissues, is confirmed as a tumor suppressor: via directly targeting MAP3K11, miR-199a-5p suppresses the proliferation and induces cell cycle arrest of A549 and H1299 cells (Li Y. et al., 2019). Another research reports that overexpression of miR-199a-5p is capable of increasing the sensitivity of A549 and H460 cells to doxorubicin (Jin et al., 2020). However, the explicit function and underlying mechanism whereby miR-199a-5p regulates NSCLC progression remain largely unknown.

Hypoxia-inducible factor-1α (HIF-1α), defined as an oxygen-regulated protein, is in marked association with cancer biology (Semenza, 2003). Of note, in NSCLC, HIF-1α is reported to be an oncogene. For example, subsequent to the knockdown of HIF-1α with siRNA, the proliferation and invasion of NCI-H157 cells were repressed but apoptosis was promoted (Qian et al., 2016). Signal transducer and activator of transcription 3 (STAT3) is also identified as a promoter for NSCLC. For instance, downregulation of STAT3 weakens the colony-forming ability and proliferation of A549 and SK-MES-1 cells (Yin et al., 2011). Nonetheless, the mechanism of HIF-1α and STAT3 hyperactivation in NSCLC has not been clarified clearly.

**Figure 1:**
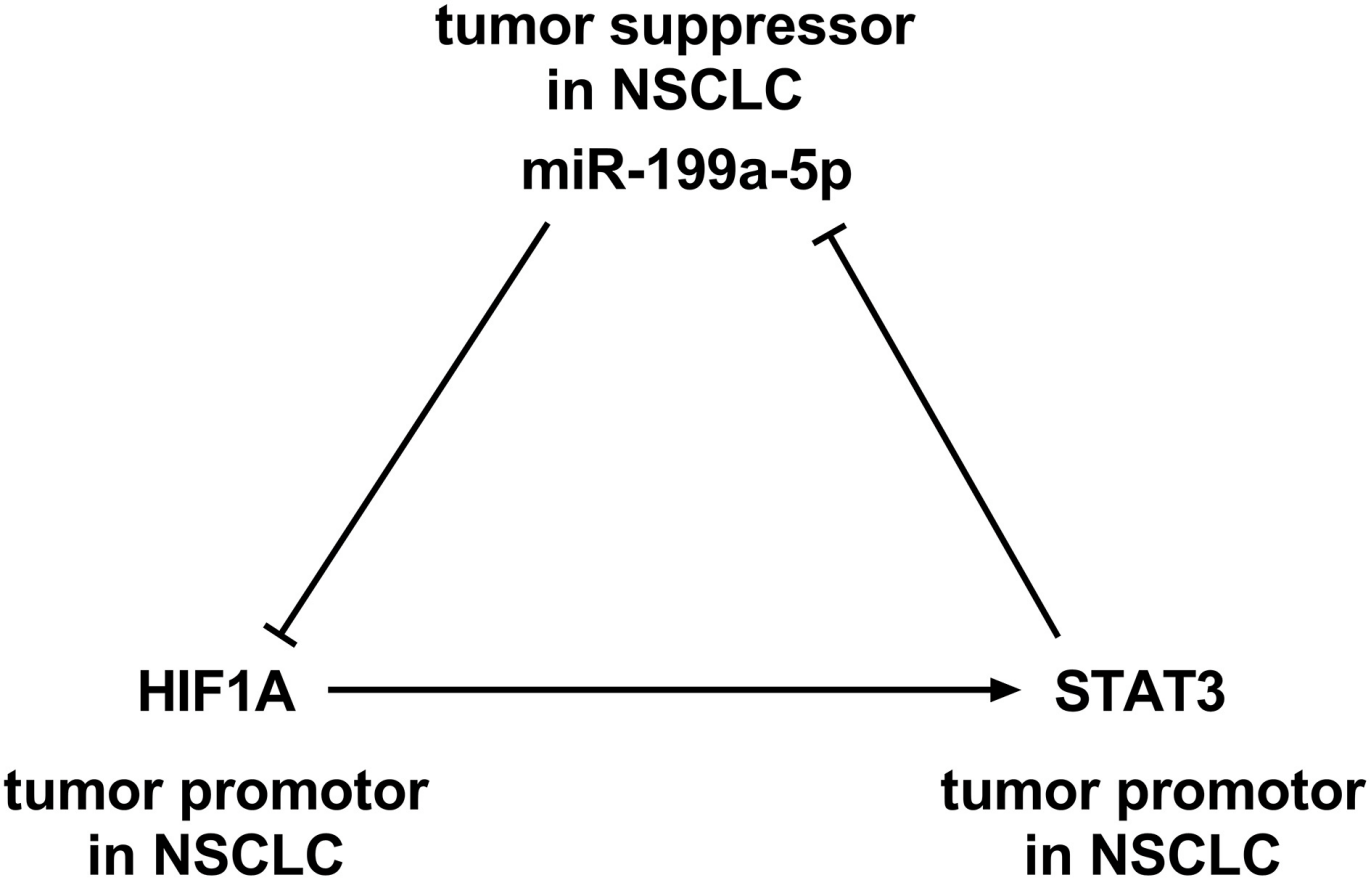
A positive feedback loop among miR-199a-5p, hypoxia-inducible factor-1α (HIF-1α), and signal transducer and activator of transcription 3 (STAT3).

Intriguingly, HIF-1α is reported to activate STAT3 by repressing miR-34a (Li et al., 2017). It is noteworthy that STAT3 knockdown in the cardiomyocyte of neonatal rat enhanced the miR-199a-5p promoter activity, and miR-199a-5p expression is demonstrably upregulated, suggesting that STAT3 negatively regulates miR-199a-5p (Haghikia et al., 2011). Besides, bioinformatic analysis implicated that potential binding sites existed between miR-199a-5p and HIF-1α. Based on these information, we supposed that miR-199a-5p–HIF-1α-STAT3 could probably be a positive feedback loop in NSCLC development (Figure 1). This study was designed to validate this scientific hypothesis.

## Materials and Methods

### Reanalysis of Gene Expression Omnibus Data

The Gene Expression Omnibus (GEO) datasets GSE135918 (Jiang et al., 2020) and GSE53882 (Pu et al., 2017) were downloaded (from https://www.ncbi.nlm.nih.gov/gds) and reanalyzed. GSE135918 contains miRNA expression profile data of five pairs of fresh lung cancer tissue specimens and non-tumor lung tissue specimens. GSE53882 contains miRNA expression profile data from 397 NSCLC tissues and 151 non-cancerous lung tissues. With the GEO2R tool, significantly differentially expressed miRNAs in cancer tissues relative to normal tissues were screened.

### Clinical Samples

Thirty patients with NSCLC who did not receive radiotherapy or chemotherapy prior to surgery at the Thoracic Cancer Center, the Sixth Affiliated Hospital of Sun Yat-sen University, from January 2017 to January 2018, were enrolled in this research. Thirty pairs of NSCLC tissues/corresponding adjacent lung tissues as well as blood samples of all subjects were obtained during the surgery. The blood samples of 30 healthy volunteers were also collected. All tumor tissue samples were immediately cryopreserved in liquid nitrogen at −196°C after the resection. The blood samples were stored at −80°C. The study was approved by the Ethics Committees of the Sixth Affiliated Hospital of Sun Yat-sen University. Written informed consent was acquired from every patient. The procedures were performed according to the principles of the Declaration of Helsinki.

### Cell Culture and Transfection

NSCLC cell lines (H1299, H157, A549, and H460) and normal human lung epithelial cells BEAS-2B were obtained from the American Type Culture Collection (ATCC, Rockville, MD, USA) or China Center for Type Culture Collection (Wuhan, China) and cultivated in RPMI-1640 medium (Gibco, Carlsbad, CA, USA) supplemented with 10% fetal bovine serum (FBS) (Gibco, Carlsbad, CA, USA) at 37°C with 5% CO_2_. Construction of miR-199a-5p mimics, control of mimics, miR-199a-5p inhibitors, control of inhibitors, overexpression plasmids of HIF1A and STAT3, and their negative control (vector) was accomplished by GenePharma (Shanghai, China). By the time of reaching 60% confluence, A549 and H1299 cells were transiently transfected with miR-199a-5p inhibitors, miR-199a-5p mimics, or the overexpression plasmids with Lipofectamine^TM^ 3000 (Invitrogen, Carlsbad, CA, USA) in conformity with the manufacturer's protocols. Transfection efficiency was validated 24 h after transfection with quantitative real-time polymerase chain reaction (qRT-PCR). For experimentally analyzing the resistance of NSCLC cells to bevacizumab, A549 cells were treated with 10 μM bevacizumab (Topscience, Shanghai, China).

### qRT-PCR

Isolation of total RNA from NSCLC tissues, blood samples, and cells was accomplished utilizing the TRIzol reagent (Invitrogen, Carlsbad, CA, USA). Reverse transcription of total RNA to cDNA was performed with reverse transcription kit (TaKaRa, Dalian, China). With the cDNA as template, qRT-PCR was performed utilizing SYBR Premix Ex Taq™ II (TaKaRa, Shiga, Japan) on ABI 7500 Fast Real-Time PCR System (Applied Biosystems, Waltham, MA, UK). The primer sequences utilized in our current research are shown in Table 1.

**Table 1:** Primers used for PCR.

**Genes**	**Primers**
*miR-199a-5p*	Forward: 5′-CAATCGCTTTCAAATAG-3′
	Reverse: 5′-CAGGAGATGCTGTCATC-3′
*U6*	Forward: 5′-CTCGCTTCGGCAGCACA-3′
	Reverse: 5′-AACGCTTCACGAATTTGCGT-3′
*HIF-1α*	Forward: 5′-TGATTGCATCTCCATCTCCTACC-3′
	Reverse: 5′-GACTCAAAGCGACAGATAACACG-3′
*β-Actin*	Forward: 5′-TTGCGTTACACCCTTTCTTG-3′
	Reverse: 5′-TGCTGTCACCTTCACCGTTC-3′

### Cell Counting Kit-8 Assay

A549 and H1299 cells were seeded into 96-well plates (2 × 10^3^ cells per well). After culturing for 24, 48, 72, and 96 h, each well was supplemented with 10 μl of Cell Counting Kit-8 (CCK-8) solution (Dojindo, Tokyo, Japan). After the CCK-8 solution was added, the cells were incubated for 1 h. Then, cell viability at each time point was evaluated by measuring the absorbance values at a wavelength of 450 nm. Next, the proliferation curve was plotted.

### Flow Cytometry Analysis

For the cell cycle analysis, A549 or H1299 cells were seeded into six-well plate (5 × 10^5^ cells/well) and cultured in a medium containing 0.2% serum for 24 h for synchronization. Subsequently, the cells were stained with propidium iodide (PI) solution (Beyotime, Shanghai, China) and were examined employing a FACS flow cytometer (BD Biosciences, San Jose, CA, USA). For the apoptosis analysis, A549 and H1299 cells were collected, washed, fixed, and permeabilized. Then, Annexin V-FTIC/PI Apoptosis Detection Kit (KeyGen, Nanjing, China) was employed to detect the apoptosis of the cells according to the manufacturer's instruction. The data were computationally analyzed with the ModFit software (BD Biosciences, San Jose, CA, USA) (for the cell cycle analysis) or FlowJo V10 software (BD Biosciences, San Jose, CA, USA) (for the apoptosis analysis).

### Transwell Assay

The pore size of the membrane of Transwell chambers (Corning, Shanghai, China) was 8 μm. The chambers were coated with Matrigel (Sigma, Shanghai, China) in the invasion assay, and no Matrigel was added during the migration assay. The lower compartment of the Transwell system was filled with 600 μl of RPMI-1640 medium containing 10% FBS, and the upper compartment contained 200 μl of the cell suspension (5 × 10^5^ cells in each well in serum-free medium). The cells were cultured for 48 h, and then the Transwell chambers were taken out. The cells that failed to migrate were removed with cotton swabs. Then, the remaining cells attached on the lower surface of the Transwell membrane were fixed with 4% paraformaldehyde for 20 min and stained with 0.5% crystal violet solution. Ultimately, five fields of each Transwell membrane were randomly selected and the numbers of stained cells were counted under a microscope.

### Dual-Luciferase Reporter Assay

The fragment of wild-type (WT) or mutant (MUT) HIF1A 3′ UTR was inserted into the pmirGLO dual-luciferase miRNA target expression vector (Promega, Madison, WI, USA). The recombinant reporter vectors were co-transfected into H1299 cells with miR-199a-5p mimics or mimics control, and then, the cells were cultured for 48 h. Luciferase activity was subsequently measured utilizing the Dual-Luciferase Reporter Gene Assay Kit (Beyotime, Jiangsu, China) according to the manufacturer's protocol. The luciferase activity of firefly was normalized to that of Renilla.

### Western Blotting

NSCLC cells in each group were harvested and washed three times with cold PBS, and 100 μl of RIPA lysis buffer (Beyotime, Shanghai, China) was added. The cells were lysed in an ice bath for 30 min to extract the total protein. The mixtures were subsequently centrifuged at 12,000 rpm for 20 min, and the supernatant was collected as the protein samples. The protein samples were denatured, separated by sodium dodecyl sulfate-polyacrylamide gel electrophoresis, and transferred onto PVDF membranes (Millipore, Bedford, MA, USA). The membranes were then blocked with 5% skimmed milk and incubated with primary antibodies [rabbit anti-STAT3 (phospho S727) antibody, 1:1,000, ab32143; rabbit anti-STAT3 antibody, 1:1,000, ab68153; rabbit anti-HIF-1 alpha antibody, 1:1,000, ab82832] at 4°C overnight. Next, the membranes were washed with TBST buffer and then incubated with secondary horseradish peroxidase (HRP)-labeled goat anti-rabbit IgG antibodies (1:2,000, ab205718, Abcam, Shanghai, China) for an additional 2 h at room temperature. β-Actin was employed as the internal reference. The protein bands on the membranes were then detected utilizing the ECL kit (Amersham Pharmacia Biotech, Little Chalfont, UK) and computationally analyzed with ImageJ software (National Institutes of Health, Bethesda, MD, USA).

### Xenograft Models

The procedures of the animal experiments have been reviewed and approved by the Animal Ethics Committee of the Sixth Affiliated Hospital of Sun Yat-sen University and are in compliance with the National Institute of Health Guidelines for the Care and Use of Laboratory Animals in Biomedical Research. A549 cells, previously reported to be resistant to bevacizumab, were selected for establishing xenograft models. Subsequent to transfection with miR-199a-5p mimics, miR-199a-5p inhibitors, HIF-1α, and STAT3 overexpression plasmid or their negative controls, A549 cells were inoculated subcutaneously into the right flank region of male athymic (BALB/c-nu) mice (6 weeks old). By the time the tumor size reached approximately 100 mm^3^, 27 mice were randomly divided into nine groups with three mice per group: blank group (no treatment), BV group (mice were injected with 10 mg/kg bevacizumab weekly), and BV+control of mimics group (A549 cells were transfected with control of mimics prior to injection and mice were injected with 10 mg/kg bevacizumab weekly). As for the rest of the groups, A549 cells were transiently transfected with different plasmids or oligonucleotides prior to injection and mice were injected with 10 mg/kg bevacizumab weekly: BV+control of inhibitors group, BV+miR-199a-5p mimics group, BV+miR-199a-5p inhibitors group, BV+miR-199a-5p mimics+vector group, BV+miR-199a-5p mimics+HIF-1α group, and BV+miR-199a-5p mimics+STAT3 group. The tumor volume was recorded every 2 days. The tumor volume (in mm^3^) was calculated with the formula 0.5 × *L* × *W*2 (*L* = length, *W* = width).

### Statistical Analysis

All statistical analyses were carried out utilizing the SPSS 23.0 software (SPSS Inc., Chicago, IL, USA). Data were presented as “mean ± standard error.” Data were examined whether they were normally distributed with the one-sample Kolmogorov–Smirnov test. As for the data normally distributed, *t*-test or one-way ANOVA test was performed. As for the data not normally distributed, rank test was conducted. Pearson's correlation analysis was used to analyze the correlations of the gene expression levels. *p* < 0.05 was deemed to be statistically significant.

## Results

### Expression of miR-199a-5p Was Downregulated in NSCLC

**Figure 2:**
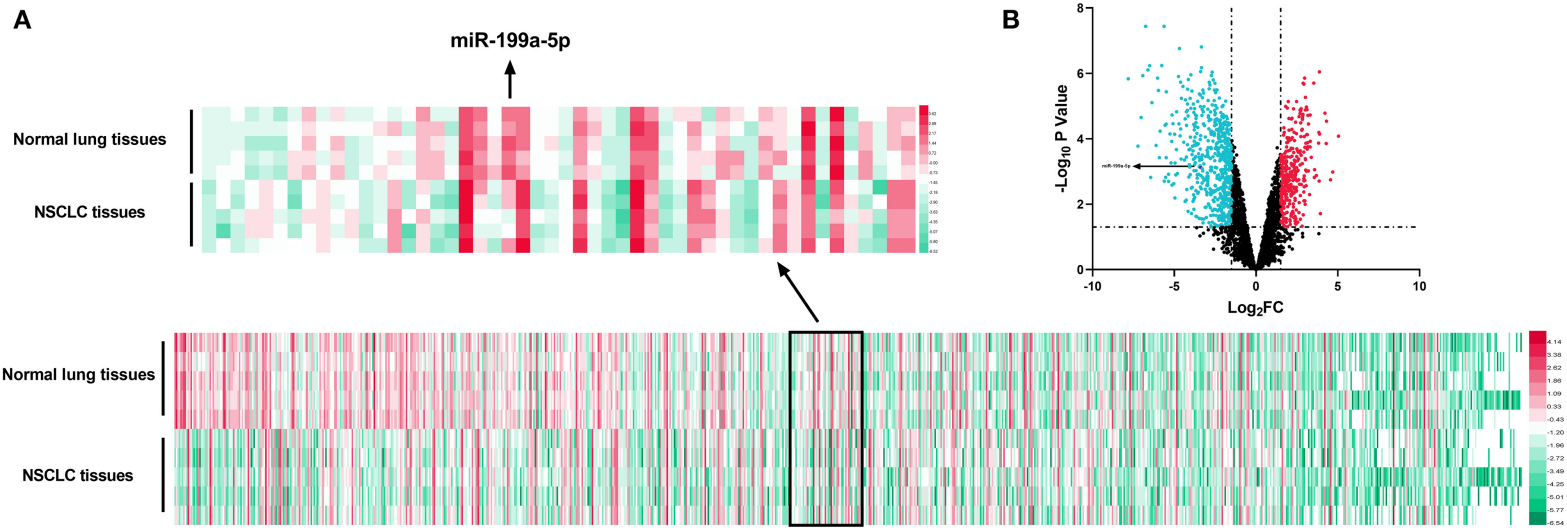
Expression of miRNAs in GSE135981. The heatmap and volcano plot were established with the data in GSE135981. **(A)** MiRNAs with significant changes of expression level between tumor tissues vs. normal lung tissues and |log_2_FC| >1.5 were shown in the heatmap. **(B)** All the miRNAs were shown in the volcano plot, and significantly upregulated miRNAs with log_2_FC > 1.5 were marked in red, while significantly downregulated miRNAs with log_2_FC < −1.5 were marked in green.

**Figure 3:**
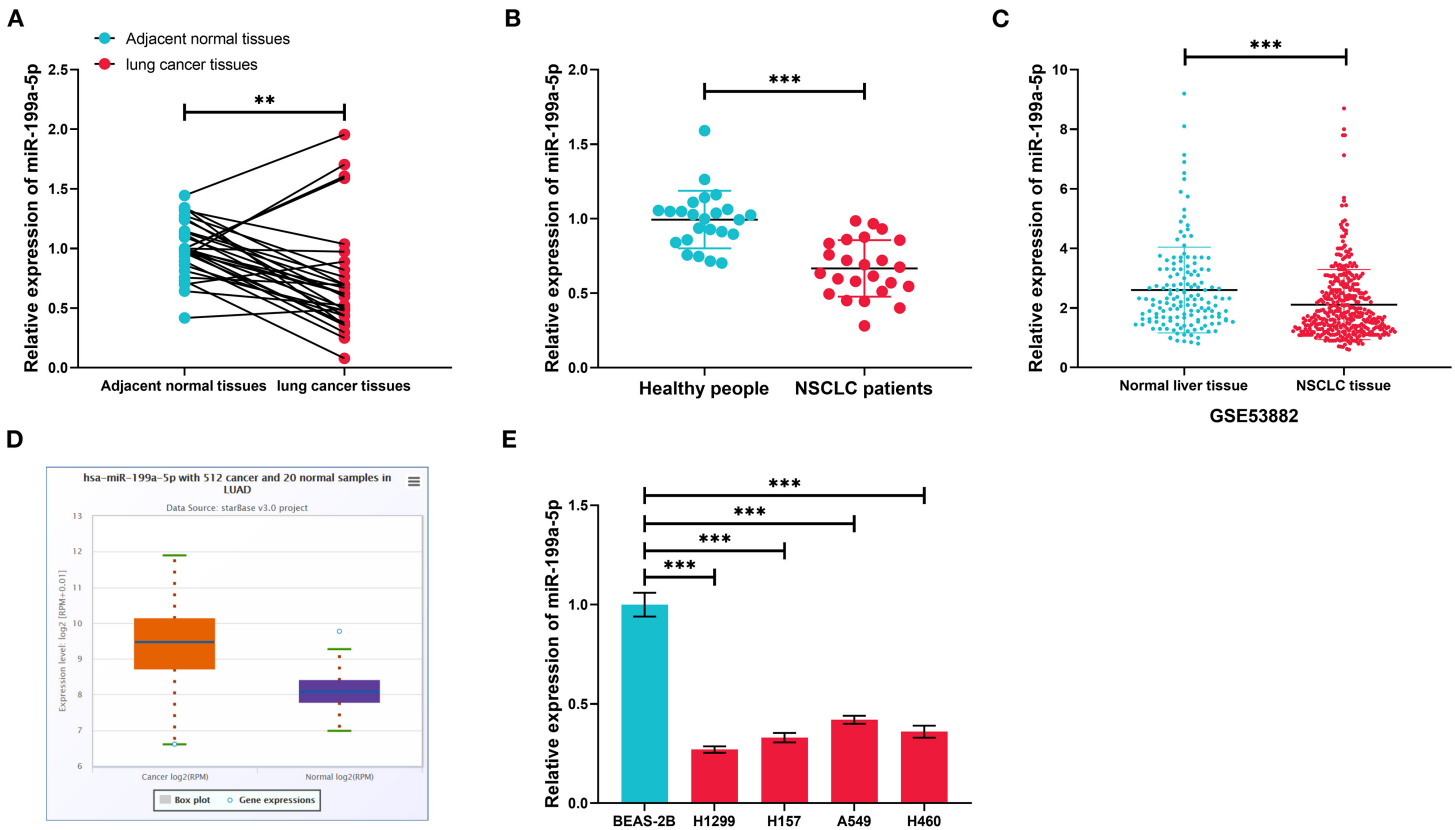
The expression characteristics of miR-199a-5p in non-small cell lung cancer (NSCLC). **(A)** Expression of miR-199a-5p in 30 pairs of NSCLC tissues and adjacent normal lung tissues was detected by qRT-PCR. **(B)** Expression of miR-199a-5p in serum samples of NSCLC patients (*n* = 30) and healthy subjects (*n* = 30) was detected by qRT-PCR. **(C)** Expression of miR-199a-5p in 397 cases of NSCLC tissues and 151 cases of normal lung tissues. The data were derived from GSE53882. **(D)** Expression of miR-199a-5p in 512 cases of lung adenocarcinoma tissues and 20 cases of normal lung tissues in the ENCORI database. **(E)** Expression of miR-199a-5p in normal lung epithelial cells and NSCLC cell lines was detected by qRT-PCR. All of the experiments were performed in triplicate. **p < 0.01 and ***p < 0.001, respectively.

GSE135918 contained the miRNA expression profile of five fresh lung cancer tissues and five fresh non-tumor lung tissues. It was reanalyzed, and it was found that miR-199a-5p was significantly downregulated in NSCLC tissues compared with normal lung tissues (Figures 2A,B). Consistently, qRT-PCR data manifested that miR-199a-5p was markedly reduced in NSCLC tissues as opposed to adjacent normal lung tissues (Figure 3A). Additionally, miR-199a-5p was observed to be reduced in the serum samples of NSCLC patients vs. healthy subjects (Figure 3B). The downregulation of miR-199a-5p in NSCLC was further validated in the GSE53882 and ENCORI database (Figures 3C,D). Furthermore, miR-199a-5p expression was demonstrated to be underexpressed in NSCLC cell lines compared with normal lung epithelial cells BASE-2B (Figure 3E).

### MiR-199a-5p Repressed the Proliferation, Migration, and Invasion of NSCLC

**Figure 4:**
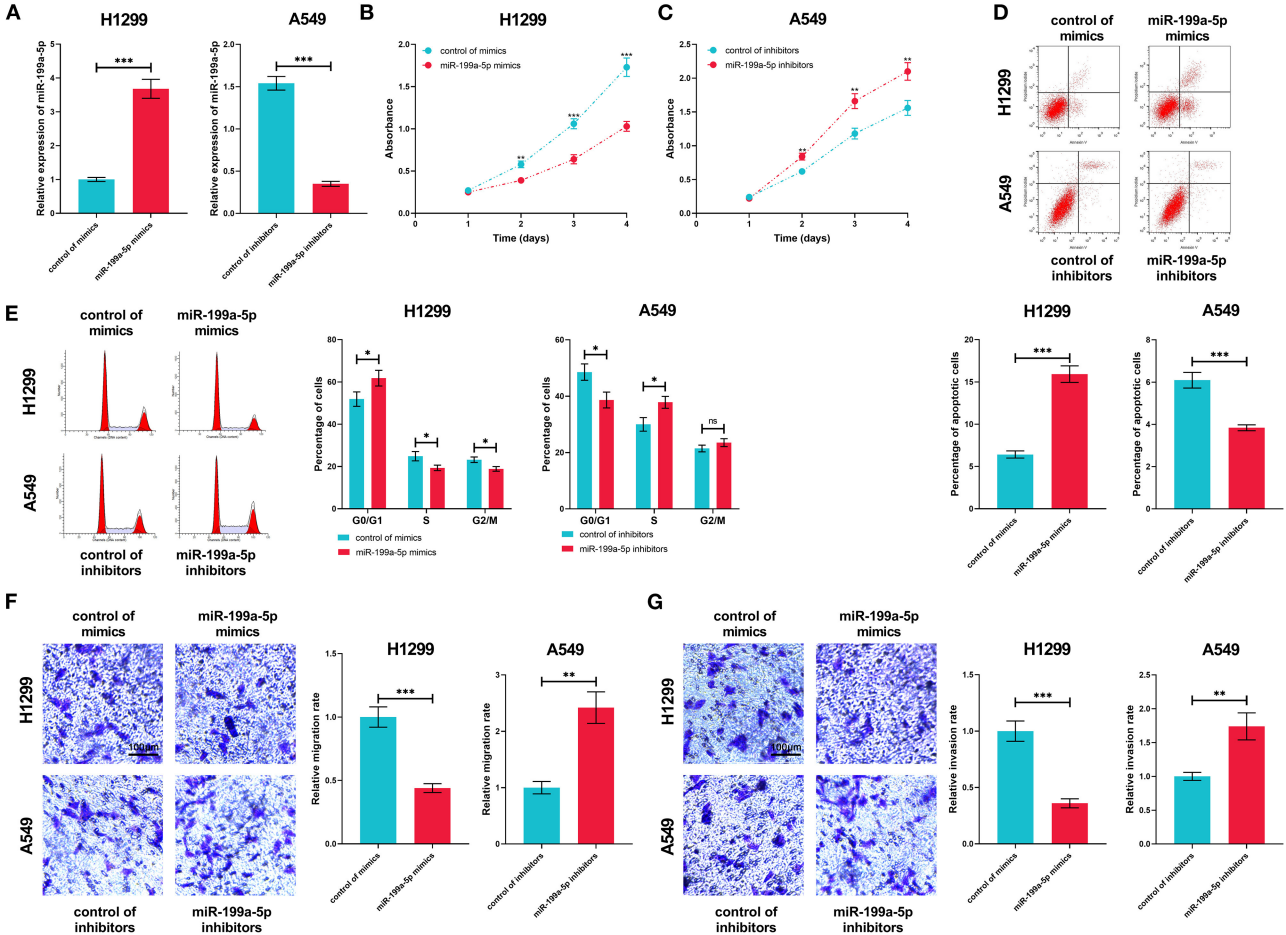
Biological function of miR-199a-5p in NSCLC. **(A)** In H1299 and A549 cells, the transfection efficiency of miR-199a-5p mimics and inhibitors was confirmed by qRT-PCR. **(B,C)** CCK-8 assay was performed to measure the proliferation of H1299 and A549 cells. **(D,E)** Flow cytometry was conducted to analyze cell cycle and apoptosis of H1299 and A549 cells. **(F,G)** The Transwell assay was employed to detect migration and invasion of H1299 and A549 cells. All of the experiments were performed in triplicate. *p < 0.05, **p < 0.01, and ***p < 0.001, respectively.

Next, miR-199a-5p mimics and miR-199a-5p inhibitors were transfected into H1299 and A549 cells, based on the fact that miR-199a-5p expression was the lowest in H1299 cells and the highest in A549 cells (Figure 4A). The CCK-8 assay indicated that miR-199a-5p overexpression repressed the proliferation of H1299 cells, while the proliferation of A549 cells was promoted after miR-199a-5p was inhibited (Figures 4B,C). Furthermore, flow cytometry analysis showed that H1299 cells were arrested in G0/G1 phase after miR-199a-5p was overexpressed, whereas miR-199a-5p inhibitors induced more A549 cells entering the S phase (Figure 4D). What's more, the apoptosis of H1299 and A549 cells was analyzed and it was demonstrated that miR-199a-5p overexpression promoted the apoptosis of NSCLC cells (Figure 4E). Additionally, Transwell assays suggested that miR-199a-5p suppressed the migration and invasion of H1299 cells, while inhibiting miR-199a-5p promoted the migration and invasion of A549 cells (Figures 4F,G). Collectively, these data validated that miR-199a-5p was a tumor suppressor of NSCLC.

### HIF-1α Was Confirmed as a Target Gene of miR-199a-5p

**Figure 5:**
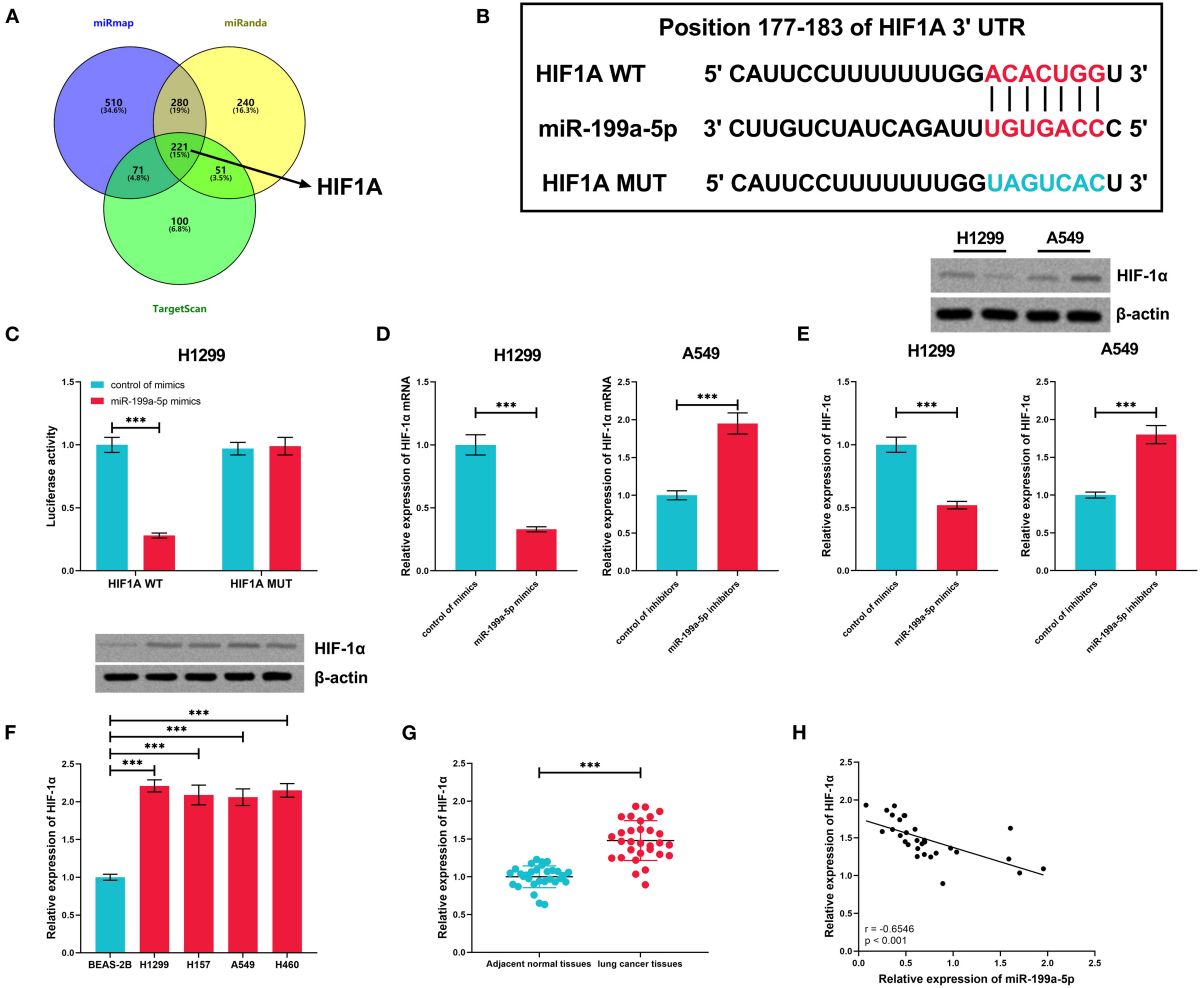
HIF1A was regulated by miR-199a-5p in NSCLC cells. **(A)** Potential downstream target genes of miR-199a-5p were co-predicted with the miRmap, miRanda, and TargetScan databases. **(B)** Conserved binding sequence between HIF1A 3′UTR and miR-199a-5p. **(C)** Dual-luciferase reporter assay was performed to verify the predicted binding site between miR-199a-5p and HIF1A 3′UTR. **(D,E)** qRT-PCR and Western blotting were conducted to measure the expression of HIF1A mRNA and HIF1A protein in H1299 and A549 cells after the transfection of miR-199a-5p mimics or inhibitors. **(F)** Western blotting was performed to measure HIF-1α protein expression in NSCLC cell lines. **(G)** Expression of HIF-1α in 30 pairs of NSCLC tissues and adjacent normal tissues was detected by qRT-PCR. **(H)** Correlation between HIF-1α expression and miR-199a-5p expression in NSCLC tissues. All of the experiments were performed in triplicate. ***p < 0.001.

The miRmap, miRanda, and TargetScan databases were searched for the downstream target genes of miR-199a-5p, and 221 genes were predicted by all of the three databases (Figure 5A). HIF1A, the gene of HIF-1α, previously reported to exert a tumor-promoting role in NSCLC (Lin et al., 2016; Qian et al., 2016), was among them (Figure 5B). Dual-luciferase reporter assay was employed for verifying the binding site, and as shown, miR-199a-5p mimics remarkably suppressed the luciferase activity of the wild-type HIF1A reporter, whereas this effect was abolished when the binding site was mutated (Figure 5C). Furthermore, via qRT-PCR and Western blotting, it was demonstrated that miR-199a-5p repressed the expressions of HIF-1α mRNA and protein in H1299 cells, while inhibiting miR-199a-5p promoted the expression of HIF-1α in A549 cells (Figures 5D,E). Besides, HIF-1α expression in NSCLC cell lines and tissues was significantly upregulated (Figures 5F,G). Moreover, the expression of miR-199a-5p in NSCLC tissues was in negative correlation with HIF1A (Figure 5H). These data confirmed the targeting relationship between miR-199a-5p and HIF1A.

### The Positive Feedback Loop Among miR-199a-5p, HIF-1α, and STAT3

**Figure 6:**
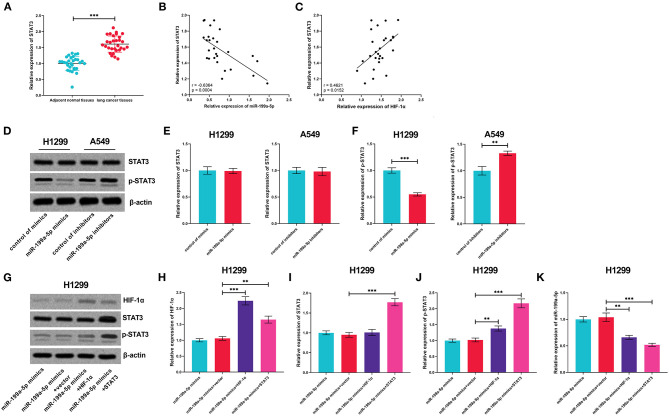
The positive feedback loop was found among miR-199a-5p, HIF-1α, and STAT3. **(A)** Expression of STAT3 in 30 pairs of adjacent normal lung tissues and NSCLC tissues was detected by qRT-PCR. **(B,C)** Correlation between STAT3 expression and miR-199a-5p expression or HIF-1α expression in NSCLC tissues. **(D–F)** Western blotting was used to detect STAT3, p-STAT3, and HIF-1α expressions in H1299 and A549 cells after the transfection of miR-199a-5p mimics or miR-199a-5p inhibitors. **(G–J)** Western blotting was used to measure the expressions of STAT3, p-STAT3, and HIF-1α in H1299 cells after miR-199a-5p, HIF-1α, and STAT3 were selectively regulated by transfection. **(K)** qRT-PCR was employed to detect miR-199a-5p expression in H1299 cells after miR-199a-5p, HIF-1α, and STAT3 were selectively regulated by transfection. All of the experiments were performed in triplicate. **p < 0.01 and ***p < 0.001, respectively.

Subsequently, the expression of STAT3 mRNA was measured by qRT-PCR, the results of which displayed that STAT3 was upregulated in NSCLC tissues, in negative correlation with miR-199a-5p but in positive correlation with HIF-1α (Figures 6A–C). Followed by miR-199a-5p overexpression or inhibition in NSCLC cells, no obvious alteration was noted in STAT3 expression, whereas miR-199a-5p was observed to inactivate STAT3 phosphorylation; besides, it was demonstrated that HIF-1α overexpression activated the phosphorylation of STAT3, while STAT3 overexpression or HIF-1α overexpression further reduced miR-199a-5p expression in H1299 cells (Figures 6D–K). Collectively, these data show that a positive feedback loop of miR-199a-5p–HIF-1α-STAT3 exists in NSCLC.

### HIF-1α and STAT3 Counteracted the Effects of miR-199a-5p on NSCLC

**Figure 7:**
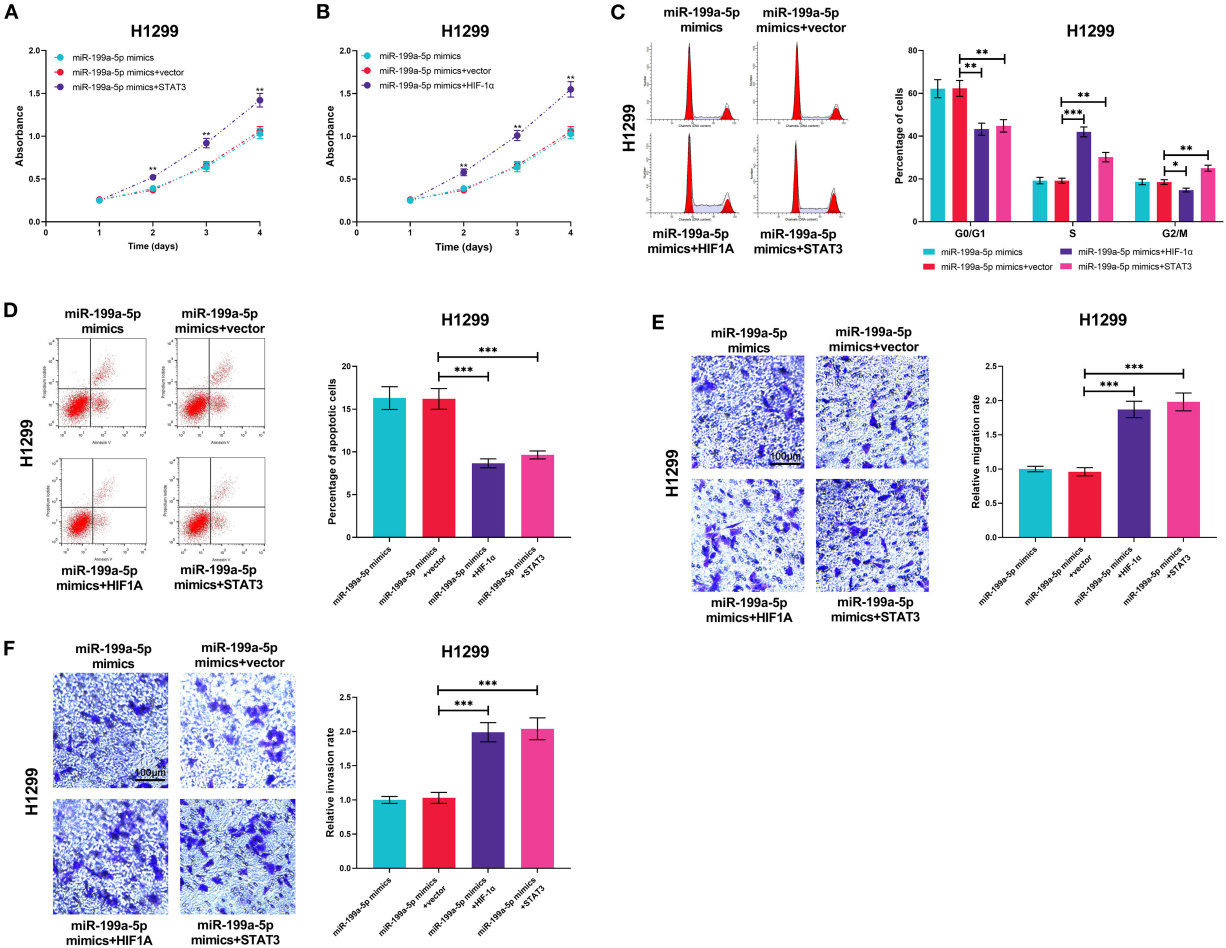
HIF-1α overexpression or STAT3 overexpression reversed the biological effects of miR-199a-5p on H1299 cells. H1299 cells were transfected with miR-199a-5p mimics or co-transfected with miR-199a-5p mimics and STAT3 overexpression plasmids (or HIF-1α overexpression plasmids). **(A,B)** CCK-8 assay was performed to measure the proliferation of H1299 cells. **(C,D)** Flow cytometry was used to detect H1299 cell cycle and apoptosis. **(E,F)** Migration and invasion of H1299 cells were analyzed employing the Transwell assay. All of the experiments were performed in triplicate. *p < 0.05, **p < 0.01, and ***p < 0.001, respectively.

MiR-199a-5p mimics and HIF-1α or STAT3 overexpression plasmids were transiently co-transfected into H1299 cells so as to further investigate the interactions among miR-199a-5p, HIF-1α, and STAT3. As expected, the effects of miR-199a-5p on proliferation, cell cycle, apoptosis, migration, and invasion were reversed by HIF-1α overexpression or STAT3 overexpression (Figures 7A–F), which supported that miR-199a-5p regulated the malignancy of NSCLC cells by modulating HIF-1α and STAT3.

### MiR-199a-5p Enhanced the Sensitivity of A549 Cells to Bevacizumab

**Figure 8:**
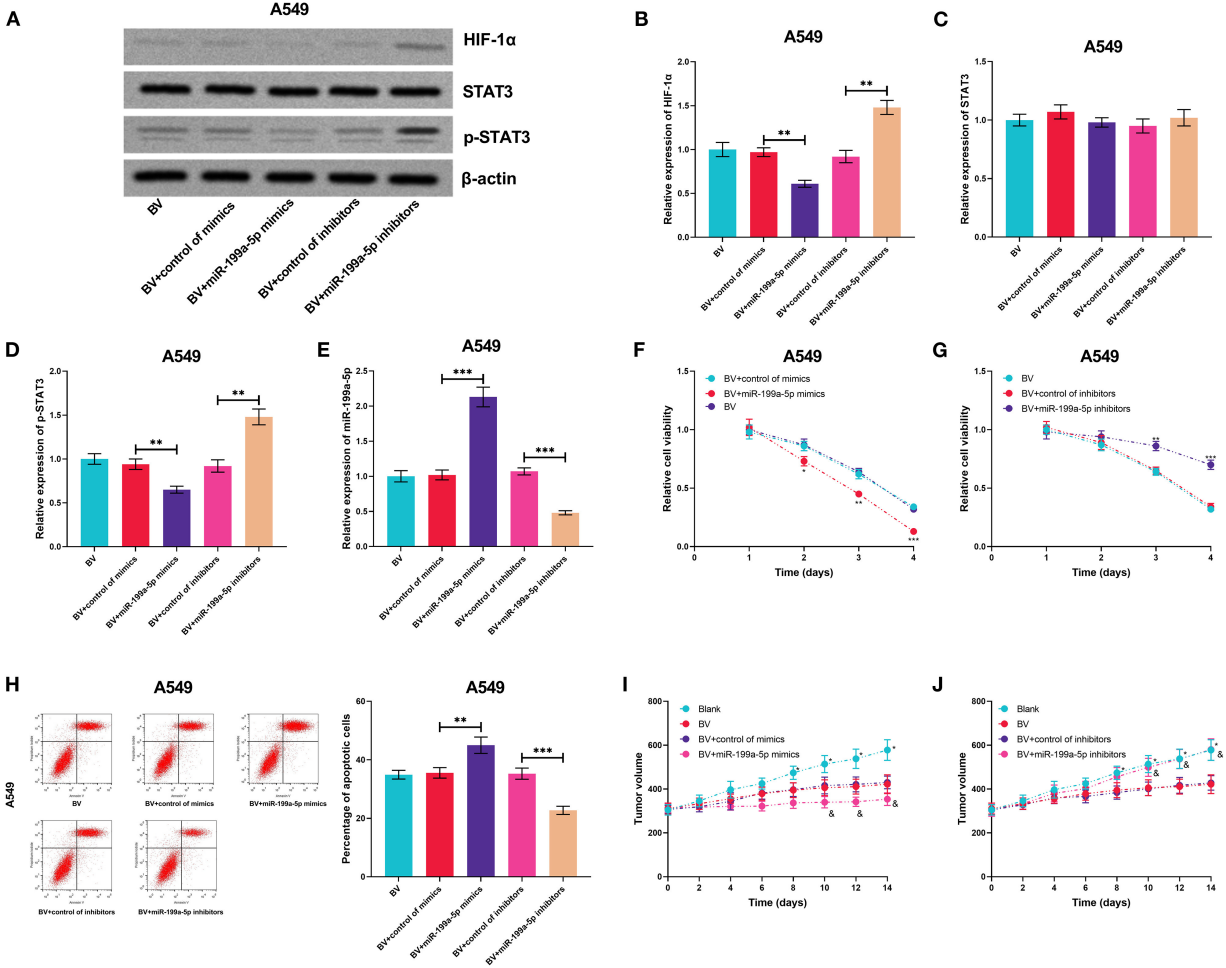
MiR-199a-5p enhanced the effects of bevacizumab on A549 cells. After A549 cells were transfected with miR-199a-5p mimics, or miR-199a-5p inhibitors, the cells were treated with bevacizumab (BV). **(A–D)** Western blotting was used to detect the expressions of HIF-1α, STAT3, and p-STAT3 in A549 cells. **(E)** qRT-PCR was used to detect miR-199a-5p expression in A549 cells after the transfection and BV treatment. **(F,G)** Proliferation of A549 cells was examined by the CCK-8 assay. **(H)** Apoptosis of A549 cells was measured using flow cytometry. **(I,J)** After A549 cells were transfected, the cells were planted into the mice, and then the mice were treated with BV. The changes of tumor volume with time in each group were recorded. All of the experiments were performed in triplicate. *p < 0.05, **p < 0.01, and ***p < 0.001, respectively. In **(I,J)**, and represents *p* < 0.05 BV+control of mimics/inhibitors group vs. BV+miR-199a-5p mimics/inhibitors group.

The high expression of HIF-1α and the activation of STAT3 signaling are reported to contribute to bevacizumab resistance (Hartwich et al., 2013; Jahangiri et al., 2013). Therefore, we supposed that miR-199a-5p could be a potential therapeutic target for sensitizing NSCLC cells to bevacizumab treatment. A549 cells, previously reported to show moderate resistance to bevacizumab treatment (Wang et al., 2016), were used for the subsequent experiments. MiR-199a-5p mimics or inhibitors were transiently transfected into A549 cells, and then the cells were treated with bevacizumab (10 μM). Next, the expressions of HIF-1α, STAT3, and p-STAT3 were measured utilizing qRT-PCR, the results of which suggested that miR-199a-5p inhibited the expression of HIF-1α and activation of STAT3 (Figures 8A–E). Through the CCK-8 assay and flow cytometry, miR-199a-5p mimics were demonstrated to remarkably inhibit the viability and promote the apoptosis of A549 cells induced by bevacizumab; conversely, miR-199a-5p inhibitors repressed the viability of A549 cells and promoted apoptosis induced by bevacizumab (Figures 8F–H). Consistently, the results of the *in vivo* experiments suggested that miR-199a-5p mimics facilitated the tumor-suppressive effects of bevacizumab, while miR-199a-5p inhibitors counteracted the tumor cytotoxicity of bevacizumab (Figures 8I,J). Collectively, these findings indicated that miR-199a-5p enhanced the sensitivity of A549 cells to bevacizumab.

### HIF-1α and STAT3 Enhanced the Resistance of A549 Cells to Bevacizumab

**Figure 9:**
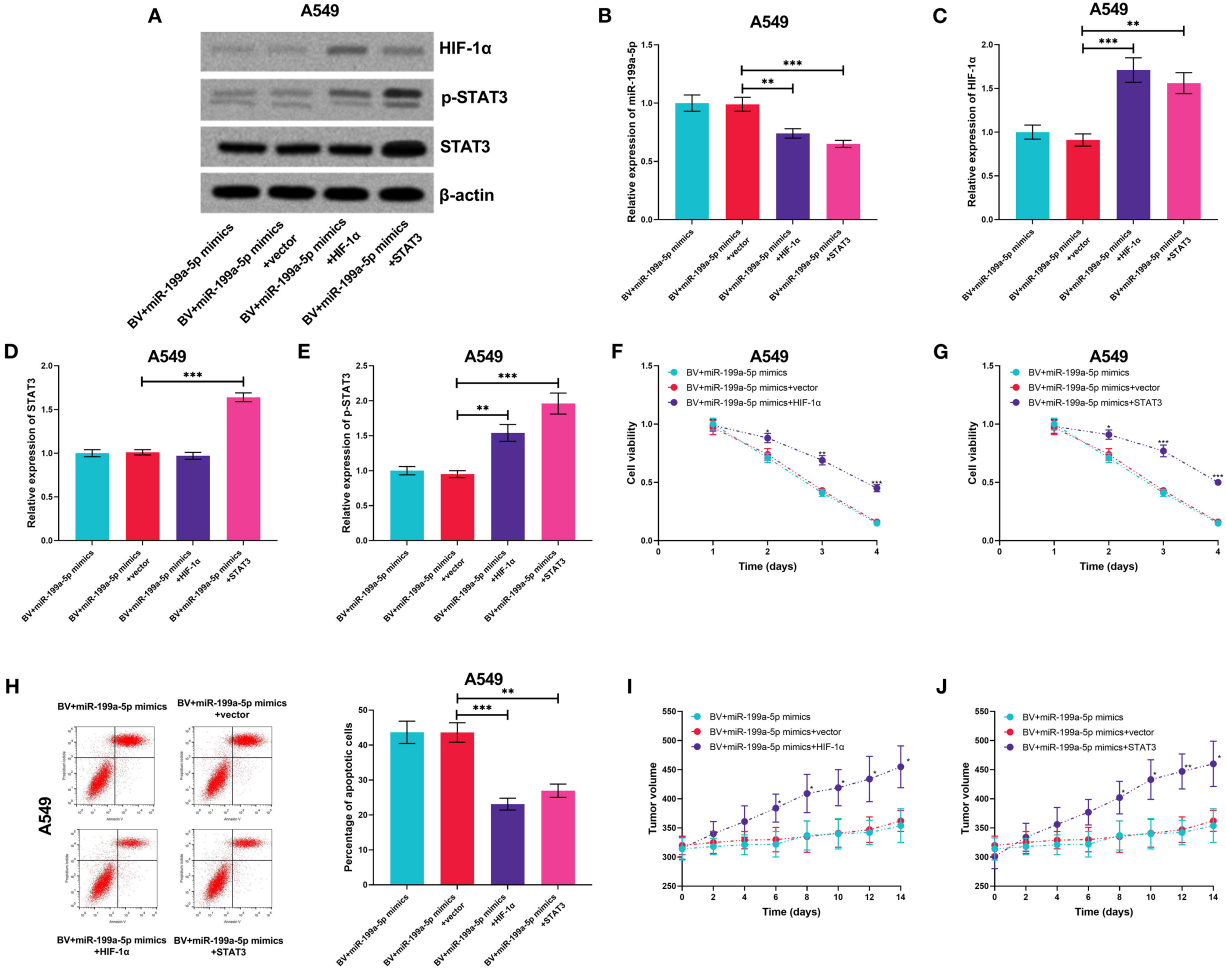
HIF-1α and STAT3 reversed the effects of miR-199a-5p on enhancing the sensitivity of A549 cells to bevacizumab. After A549 cells were transfected with miR-199a-5p mimics, or co-transfected with miR-199a-5p mimics and HIF-1α overexpression plasmids (or STAT3 overexpression plasmids), the cells were treated with bevacizumab (BV). **(A–D)** Western blotting was used to detect the expressions of HIF-1α, STAT3, and p-STAT3 in A549 cells. **(E)** qRT-PCR was used to detect miR-199a-5p expression in A549 cells after the transfection and BV treatment. **(F,G)** Proliferation of A549 cells was examined by the CCK-8 assay. **(H)** Apoptosis of A549 cells was measured using flow cytometry. **(I,J)** After A549 cells were transfected, the cells were planted into the mice, and then the mice were treated with BV. The changes of tumor volume with time in each group were recorded. All of the experiments were performed in triplicate. *p < 0.05, **p < 0.01, and ***p < 0.001, respectively.

For further deciphering whether miR-199a-5p increased the sensitivity of A549 cells to bevacizumab by suppressing the expression of HIF-1α and the activation of STAT3, miR-199a-5p mimics and HIF-1α or STAT3 overexpression plasmids were co-transfected into A549 cells (Figures 9A–E). It was experimentally demonstrated that both HIF-1α and STAT3 were capable of remarkably eliminating the effects of miR-199a-5p on the sensitivity of A549 cells to bevacizumab *in vitro* and *in vivo* (Figures 9F–J). These data validated that miR-199a-5p was capable of sensitizing A549 cells to bevacizumab through regulating HIF-1α and STAT3.

## Discussion

Multiple miRNAs have been identified as potential diagnostic biomarkers and therapeutic targets for NSCLC. For example, the expression of miR-128-3p is significantly upregulated in NSCLC tissues in comparison with adjacent normal tissues; its overexpression remarkably induces the migration and invasion of A549, Calu-3, and H520 cells (Weidle et al., 2019). In the present work, miR-199a-5p was found to be significantly downregulated in NSCLC tissues, serum samples of NSCLC patients, and multiple NSCLC cell lines. Gain-of-function and loss-of-function experiments further illustrated that miR-199a-5p repressed the proliferation, migration, and invasion and facilitated the apoptosis of NSCLC cells, indicative of a prospective value of miR-199a-5p in NSCLC therapeutics. Some other studies also demonstrate that miR-199a-5p represses the malignancy of NSCLC cell lines, such as H1299, A549, and SPCA-1 cells, which is consistent with our demonstrations (Wang et al., 2018; Hua et al., 2019). The underexpression of miR-199a-5p in NSCLC has been reported by several previous reports (Wang et al., 2018; Hua et al., 2019; Li Y. et al., 2019; Jin et al., 2020). The “competitive endogenous RNA (ceRNA)” mechanism can probably contribute to the dysregulation of miR-199a-5p in NSCLC. It is reported that LINC01123 sponges miR-199a-5p as a ceRNA to negatively regulate its expression in NSCLC cells (Hua et al., 2019); similarly, in NSCLC cells, miR-199a-5p is reported to be adsorbed and repressed by lncRNA PVT1 (Wang et al., 2018). Interestingly, in cardiomyocytes, p-STAT3 is able to bind to the promoter region of the miR-199a-2 gene and can repress its transcription and downregulate its expression, which is more significant under hypoxic conditions, suggesting that miR-199a-5p is modulated by STAT3 signaling and HIF-1α signaling (Haghikia et al., 2011; Zhou et al., 2019). In the present study, for the first time, we demonstrated that overexpression of STAT3 or HIF-1α decreased the expression of miR-199a-5p in NSCLC cells, which indicated that the hypoxic tumor microenvironment and activation of STAT3 signaling contributed to the dysregulation of miR-199a-5p in NSCLC cells.

HIF-1α, recognized as oxygen-labile subunit of HIF-1, contains the transactivation domains responsible for the transcriptional activity of HIF-1 (Albadari et al., 2019; Tirpe et al., 2019). Despite the fact that HIF-1α is merely highly expressed during hypoxic condition, it is also detectable in normoxic condition. Hypoxia is a common characteristic in the microenvironment of multiple solid tumors, promoting HIF-1α expression (Gu et al., 2015; Masoud and Li, 2015; Albadari et al., 2019). In turn, HIF-1α is conducive to the adjustment of tumors to hypoxia through transcriptional activation of more than 100 downstream genes including LEP, EPO, PKM, etc.; in this regard, HIF-1α further promotes the proliferation of tumor cells (Masoud and Li, 2015). In NSCLC, HIF-1α is upregulated in tumor tissues and cell lines, and its overexpression facilitates the proliferation, migration, and invasion of cancer cells; what's more, the 5 year survival rate of patients with a low expression level of HIF-1α is higher than that of patients with a high expression level of HIF-1α (He et al., 2016; Jing et al., 2017; Chi et al., 2019). To shed more light on the underlying mechanism by which miR-199a-5p exhibited its tumor-suppressive role in NSCLC, the miRmap, miRanda, and TargetScan databases were employed for predicting the target genes of miR-199a-5p in this study. Interestingly, the gene of HIF-1α, HIF1A, was validated as a target gene of miR-199a-5p in NSCLC in the present study. Our data suggested that the dysregulation of HIF-1α in NSCLC is not only due to the hypoxic microenvironment but also partly from the downregulation of upstream miRNAs.

STAT3, a member of the signal transducer and activator of transcription (STAT) family, regulates the expressions of genes relevant to cell cycle, cell survival, and immune response. In recent years, STAT3 has been found to be constitutively activated in multiple types of human cancers, indicating that STAT3 is a valuable target for cancer therapy (Furtek et al., 2016). STAT3 has been identified to be abnormally increased in NSCLC tissues and cell lines, and knockdown of STAT3 can induce apoptosis and reduce the proliferation, migration, and invasion of A549 and H1975 cells (Li Z. Y. et al., 2019). Consistently, we demonstrated that transfection of STAT3 overexpression plasmids notably induced the proliferation, migration, and invasion of NSCLC cells. It is noteworthy that HIF-1α is previously reported to activate STAT3 by repressing miR-34a in colorectal cancer (Li et al., 2017). Furthermore, in head and neck squamous cell carcinoma, with inhibition of STAT3 activation, HIF-1α expression is repressed (Adachi et al., 2012). In the present work, we demonstrated that HIF-1α overexpression activated STAT3, and STAT3 overexpression repressed the expression of miR-199a-5p but promoted the expression of HIF-1α. Altogether, our study presented a novel positive feedback loop, which was formed by miR-199a-5p, HIF-1α, and STAT3 in NSCLC, and this positive feedback loop is crucial to clarify the mechanism of hyperactivation of the HIF-1α pathway and STAT3 signaling in NSCLC tissues.

At the last part of our present study, we analyzed the sensitivity of A549 cells to bevacizumab and found that a positive feedback loop also contributed to regulating the bevacizumab sensitivity of NSCLC cells. Bevacizumab, possessing high efficacy and safety, is a monoclonal anti-VEGF antibody and an encouraging target drug for NSCLC, particularly for those patients with advanced NSCLC (Besse et al., 2015; Assoun et al., 2017). There is a limited study on the molecular mechanism regarding bevacizumab sensitivity in NSCLC in the past. Herein, with the results of *in vitro* and *in vivo* experiments, we reported that the miR-199a-5p–HIF-1α-STAT3 positive feedback loop also regulated the sensitivity of NSCLC cells to bevacizumab treatment. Our study provides a new thought to further improve the clinical outcomes for bevacizumab therapy.

In aggregate, our research confirms a positive feedback loop among miR-199a-5p, HIF-1α, and STAT3 in NSCLC. The loop contributes to NSCLC progression and resistance to bevacizumab. Our results help clarify the mechanism of NSCLC tumorigenesis and progression and provide useful clues for NSCLC therapy.

## Data Availability Statement

The original contributions presented in the study are included in the article/supplementary material, further inquiries can be directed to the corresponding author/s.

## Ethics Statement

The procedures of animal experiments have been reviewed and approved by the Animal Ethics Committee of the Sixth Affiliated Hospital of Sun Yat-sen University and in compliance with the National Institute of Health Guidelines for Care and Use of Laboratory Animals in Biomedical Research.

## Author Contributions

HL conceived and designed the experiments and the structure of the manuscript. XY, YZ, JT, and WC performed the experiments. XY, YZ, RT, and PS wrote the original manuscript. JT and RT performed the statistical analysis. XY, YZ, and HL revised the manuscript. All authors read and approved the submitted version.

## Conflict of Interest

The authors declare that the research was conducted in the absence of any commercial or financial relationships that could be construed as a potential conflict of interest.
